# Correlation between Microleakage and Absolute Marginal Discrepancy in Zirconia Crowns Cemented with Four Resin Luting Cements: An In Vitro Study

**DOI:** 10.1155/2016/8084505

**Published:** 2016-09-18

**Authors:** Abad-Coronel Cristian, Li Jeanette, Martínez-Rus Francisco, Pradíes Guillermo

**Affiliations:** Department of Buccofacial Prostheses (Stomatology I), Faculty of Dentistry, Complutense University of Madrid, Madrid, Spain

## Abstract

*Objectives*. To evaluate microleakage and absolute marginal discrepancy (AMD) and to assess correlation between AMD and microleakage with four resin luting cements.* Material and Methods*. 20 extracted human third molars were prepared for full-coverage crowns. 20 zirconia copings were made (LAVA, 3M ESPE) and cemented. Specimens were randomly allocated for each used type of cement into 4 groups, RelyX® (Rx), Multilink® (Mk), PANAVIA 2.1® (P), and Maxcem® (Mx) and immersed in 10% safranin for 72 hours. 20x magnification lenses were used to observe microleakage areas (*μ*m^2^) and images software was used to measure AMD areas (*μ*m). Discrepancy and microleakage between the cements were compared with one-way ANOVA test with confidence interval of 95%.* Results*. Rx Group showed microleakage has lowest value and AMD has highest value. P Group showed microleakage has the highest value and Mk Group presented AMD has lowest value. There were no significative differences between the cements. There were no linear correlations between microleakage and AMD; however a complex regression statistical model obtained allowed formulating an association between both variables (microleakage = AMD^0,896^).* Conclusions*. No significative differences were found among 4 types of cements. No linear correlations between AMD and microleakage were found.* Clinical Significance*. AMD is not easily related to microleakage. Characteristics of cements are fundamental to decreasing of microleakage values.

## 1. Introduction

For the long-term success of restorations with all ceramic crowns, the clinician should consider several factors. Marginal fit of prosthetic crowns is an essential requirement to achieve the goal [1]. Marginal fit discrepancy can cause plaque accumulation, secondary caries, and periodontal inflammation [2]. Factors like increased depth preparation seem to cause a bigger marginal gap [3]. Microleakage is the penetration of substances, such as bacteria, oral fluids, molecules, and/or ions, into a gap or a structural defect that is naturally present or that occurs between restorative materials and tooth structure [4]. The ADA's (American Dental Association) number 8 specification [5] suggests a maximum cement thickness of 40 *μ*m, but this range is seldom achieved [1]. Classical study of McLean and von Fraunhofer [6] stated that a maximum of 120 *μ*m is clinically tolerable. With new manufacturing techniques, that discrepancy implies a greater challenge for the new materials to seal it. Nawafleh et al. [7] stated there is no conclusive evidence about an optimum fit of contemporary systems, with a diverse range between 7.5 and 206.3 *μ*m. The applied force to adapt a crown is other important concern about the marginal fit; less than 10 N is insufficient and 100 N is excessive and may cause pulpal damage; Goracci et al. [8] established that the interfacial strength and adaptation of self-adhesive cement are enhanced when a greater force than the digital pressure (20 N) is maintained during the initial self-curing period. The marginal discrepancy measure is another key factor to be considered. Holmes et al. [9] suggested the terminology to use in order to clarify misfit and explain that standardization is probably not possible. The absolute marginal discrepancy (AMD) is the best index to determine the vertical and horizontal marginal misfit. It is the combination of the marginal gap (measurement between the axial wall of the tooth preparation and the margin of the crown) and the position of the margin that could be overextended or underextended [9]. Furthermore, technique of manufacturing crowns as CAD-CAM, cast, and slip ceramics may influence the marginal misfit [10]. Several authors [11–13] agree that microfiltration is associated with marginal discrepancy when there is a gap in which the largest amount of cement is more likely to be solubilized, after which microfiltration is present. The cementing agent plays an important role so that the characteristics and properties of the cements are important to prevent microleakage and achieve a proper marginal fit. Evidence shows that resin cements have low values of microleakage and better thixotropic capacity, diminishing the marginal misfit and better adjustment of the restoration compared with zinc phosphate cements [13]. However, they have universal applications, but the polymer degradation along the clinical time is still an issue [14]. In another study, resin-modified glass-ionomer demonstrated better marginal fit but greater microleakage than MDP-based and self-adhesive dual-cure resin cements [15].

This study aims to evaluate microfiltration and AMD and to associate level AMD with microfiltration between 4 types of resin luting cements. The null hypotheses established were as follows: (1) no differences would be found in the microleakage values between the 4 types of resin cements; (2) no differences would be found in the AMD values between the resin cements used in this study; and (3) there is no correlation between microleakage and AMD.

## 2. Materials and Methods

### 2.1. Specimen Preparation

A total of 40 freshly extracted noncarious human third molars were selected for this study. All pieces were stored in physiologic saline solution during two weeks at room temperature. Each tooth was mounted for preparations, very high viscosity type addition curing silicone (Express 2, 3M ESPE, St. Paul, MM, USA) leaving the clinical crown exposed.

Occlusal and proximal reductions were performed with 1.5 mm depth orientation grooves made with a cylindrical diamond bur (Ref. 836-012, KOMET, Gebr. Brasseler GmbH & Co. KG, Lemgo, Germany). The preparation and chamfer finishing line were concluded with a rounded diamond bur (Ref. 879-012, KOMET). Buccal and palatal walls were reduced with a round-end diamond bur with 1 mm of depth. A 6-degree tapered preparation was achieved. The angles were rounded with a medium-grained flame shaped diamond bur (Ref. 368-023, KOMET). The preparations were finished with a fine-grained bur (Ref. 368EF-016, KOMET). Prepared teeth were mounted in type IV stone (Vel-mix, Kerr, Romulus, MI, USA; lot no. 3-22295), leaving the core exposed three millimeters under the finish line. Impression making was done with putty and light body addition silicone (Express, 3M ESPE, St. Paul, MM, USA). Allocation was randomized with the IPhone Application, RandNum, (developed by Ramón Urquiza, Spain), which originated numbers to each type of luting resin cement. Each test box was codified with one to five numbers and the first letter of the respective cement. The four groups were Group Rx (RelyX Unicem, 3M ESPE, Seefeld, Germany), Group MK (Multilink, Ivoclar Vivadent, Schaan, Liechtenstein); Group P (PANAVIA, Kuraray, Osaka, Japan); Group Mx (Maxcem, Kerr, USA). Each tooth was named and 20 experimental dies were obtained. 20 zirconium oxide all ceramic crowns were elaborated with CAD/CAM technique LAVA System (3M ESPE, St. Paul, MM, USA) in the dental Lab (Prótesis S.A., Madrid, Spain).

### 2.2. Crowns Cementation

All crowns were cemented by the same operator at room temperature (22°C), strictly following the manufacturer's instructions, using 4 types of resin luting cement: PANAVIA 21, Multilink (dual resin cements), RelyX Unicem, and Maxcem (self-etch and self-adhesive cements). RelyX Unicem (lot: 376465) was supplied in predosed capsules and activated with a vibrated mixing machine (Cap-Mix, 3M ESPE, Seefeld, Germany), during 15 seconds. Then, the cement was applied into the internal surface of the crown, without previous preparation. Excesses of cement were removed with a standard exploration probe and polymerization light was applied for 20 seconds. PANAVIA 21 (lot: 041352) was mixed on a mixing block, with a base-to-catalyst ratio paste of 1 : 1, during 30 seconds. Previously, the tooth surface was etched with phosphoric acid for 30 seconds, rinsed, and dried. Liquids A and B were mixed during 5 seconds and applied to the teeth surface during 60 seconds. Luting cement was applied to the internal surface of the crown and was seated, with a light pressure during 1 minute. Then, the cement excess was removed. An oxygen blocking gel (Oxyguard II, Kuraray, Osaka, Japan) was applied during 3 minutes in the margin of the crown. Polymerization light was applied for 40 seconds. Multilink (lot: M00809) and Maxcem (lot: 4476047) were used on the dried and cleaned tooth surface; a polymerization light was applied during 2 seconds on each face of the margin of the crown. Then, 40- and 20-second periods were used for the entire crown, respectively. To exercise the same pressure a gag was designed and manufactured in the Research Assistance Unit of Universidad Complutense de Madrid.

This device consisted of a special clamp with two parts connected by two sidebars. In the upper part a central screw pressed the box against the bottom part. The head of the central screw fitted with a screwdriver adjustable chassis dynamometer with a scale (Ref. 6973, Bahco, Spain). After verifying that crowns were fully adjusted to their respective cover a force of 30 N/cm^2^ was applied by means of a screwdriver chassis dynamometer and then the crowns were cemented. Once all the crowns were cemented, the specimens were immersed at 10% safranin during 72 hours and rinsed with water. The tooth was mounted over plastic blocks, numbered, and marked with the initial letters of the respective cement in order to be correctly identified. The resin blocks were cut with disk along buccolingual direction with a water-cooling saw (Micromet, Evolution Remet, Bologna, Italy). The specimens were highly polished with a machine (LS2 Remet, Bologna, Italy) and progressive abrasive disks: numbers 800 (320 *μ*m), 1200 (190 *μ*m), and 2000 (100 *μ*m) to obtain smooth surfaces for examination.

### 2.3. Specimen Analysis

**Figure 1:**
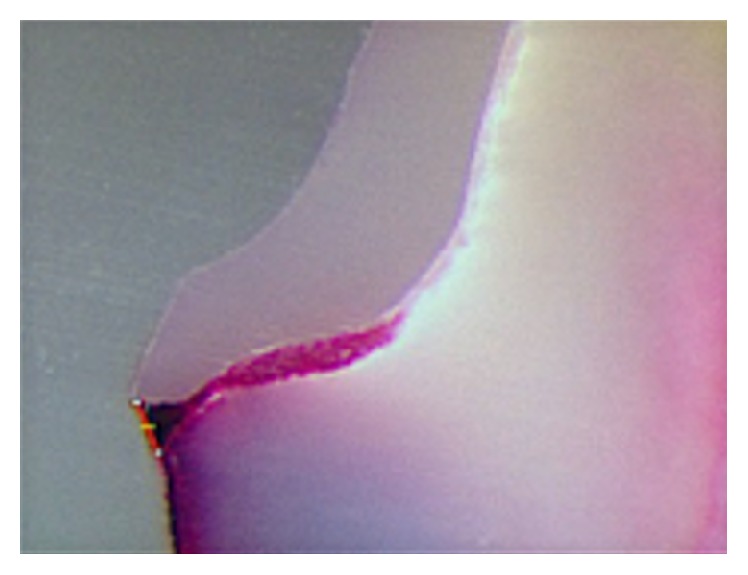
AMD image obtained (magnification lens 20x).

The 20x magnification lens (Leica Microsystems, DFC 450, Germany) analyzed cement zone stained by 10% safranin and the software (Leica Application Suite 4.0.0) delimited and measured this stained zone as microleakage in *μ*m^2^. After the cementation, AMD was defined as the maximum distance between the margin of the restoration margin and the margin of preparation (Figure 1).

### 2.4. Statistical Analysis

The maximum, minimum, and mean values of AMD and microleakage were calculated using SPSS ver. 19.0 for Mac software (SPSS IBM, Inc., Chicago, Illinois, USA). Previous Wilcoxon rank tests were performed to assess if the localization of cut of the samples influenced the AMD and microleakage value obtained and, after checking for normality, *t*-test was used to determine if the groups were different from each other according to zones of microleakage and AMD. These zones were compared in function of utilized cements with a one-way ANOVA test. In order to explain quantitatively the influence of AMD in the microleakage values we proposed a definitive statistical model with a regression analysis. *p* value was established at *p* < 0.05.

## 3. Results

**Figure 2:**
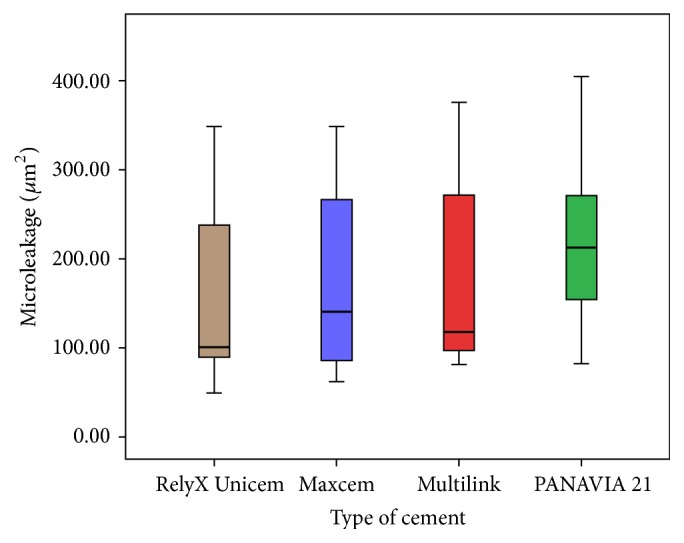
Microleakage areas (*μ*m^2^) values for each cement group. RX (brown); Mx (purple); Mk (red); and P (green).

**Figure 3:**
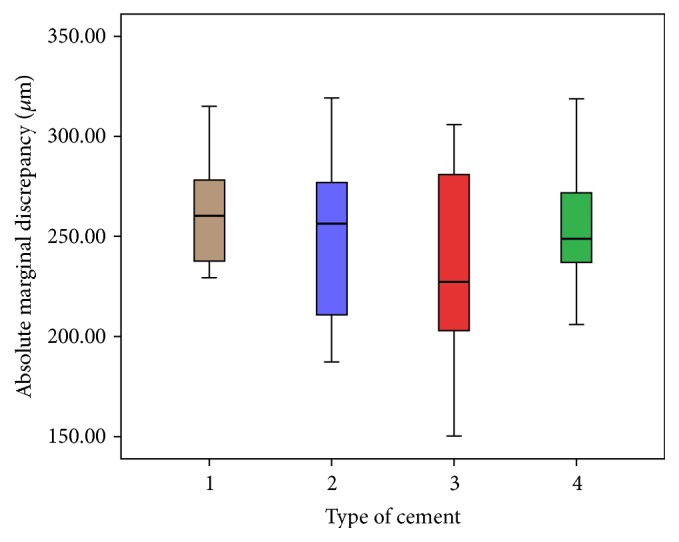
AMD values (*μ*m) for each type of cement. Rx (brown); Mx (purple); Mk (red); and P (green).

Wilcoxon rank test showed there no were statistical differences (AMD: *z* = −1.195, *p* = 0.232; microleakage: *z* = −1.157, *p* = 0.247) related to the localization of cut of the samples, and then the variables were grouped. Microleakage areas were measured (*μ*m^2^) and determined by the software according to the cement group used; RX group showed the lowest means value, followed by Mx and Mk groups (Figure 2). P group showed the highest microleakage value and AMD values were the highest for Rx Group, followed by P and Mx Groups. Mk Group showed the AMD lowest value (Figure 3). ANOVA test showed no statistical differences between the groups (Table 1).

## 4. Discussion

Obtain the best marginal fit fundamental in order to get successfully clinical longevity of dental restorations. Irregular stress concentration, secondary caries, and periodontal inflammation are possible consequences of clinical poor sealed and misfit restorations [7].

### 4.1. Microleakage

In this study, microleakage values were expressed considering the surface of extension of the cement stained with safranin (*μ*m^2^). This compared to linear measurements and numerical scores submitted would have less subjective results. The smallest value was observed in Rx Group (146 *μ*m^2^), and the highest value was from P Group (252 *μ*m^2^). These results agree with those found by Piwowarczyk et al. [16] and Hooshmand et al. [17] who measured linearly the microleakage (*μ*m). They found similar results with RelyX Unicem that exhibited the smallest value of microleakage at both tooth-cement and cement-crown interfaces. The biggest amount of microleakage was found for PANAVIA F 2.0 resin cement at both interfaces. RelyX Unicem is self-adhesive cement and requires acid etching to enhance values; PANAVIA requires more steps and is more sensitive technique. Wiedig et al. [18] found the highest adhesion values in enamel, dentin, and zirconia for RelyX compared with three other resin cements. This material showed highest ph value (6.5) that contributes to the longevity of cementation. This finding agrees with the conclusion of the studies by de Souza Costa et al. [19] and Câmara [20]. In the current study, the use of isolation in the roots was omitted and the dye penetrated into the axial and occlusal walls; this fact was interpreted by Wilson and Stankiewicz [21] as a signal of dentinal penetration.

In this study all the cements were applied in the internal walls of the zirconia copings. In other studies, Kious et al. compared the means of film thicknesses of 6 luting cements (3 of them included in this study: RelyX Unicem, Maxcem, and PANAVIA 21) at 2-minute interval after the start of mixing and none had an excessive thickness (more than 25 *μ*m) according to the ISO 9917:2 standard and it shows unnecessary extra cement space for resin cements [22] and Geerts et al. [23] did not find correlation between cement thickness and the degree of microleakage.

### 4.2. Absolute Marginal Discrepancy (AMD)

The AMD value for Rx Group was the highest (264 *μ*m) and Multilink (242 *μ*m) had the lowest one. These findings were similar to Hooshmand et al. [17] and Piwowarczyk et al. [16] data. The torch force applied in this study was 30 N. Probably the AMD values would be lower with more torch [8] due to thixotropic characteristics of the resin cements. The applied force to adapt a crown is other important concern about the marginal fit; less than 10 N is insufficient and 100 N is excessive and may cause pulpal damage; Goracci et al. [8] established that the interfacial strength and adaptation of self-adhesive cement are enhanced when a greater force than the digital pressure (20 N) is maintained during the initial self-curing period. This study did not aim to measure AMD before the cementation and showed an increasing value of 10 *μ*m after the cementation and could further influence the higher AMD value obtained. Vertical discrepancy was highest in Rx Group, probably due to its higher filler content (72%) and larger particle size (9.5 *μ*m) compared with M Group (40% filler and 0.9 *μ*m size particle) that showed the lowest value. The filler content (glass, silica) in resin cement increases the viscosity, reduces flow [24], and can affect the definitive crown seating [23]. Crowns used in this study were elaborated with CAD/CAM technology. This technology has produced dental crowns with acceptable marginal fit [10].

### 4.3. Association between Microleakage and AMD

In the current study, no linear association between microleakage and AMD was found. Both of the variables are related to the characteristics of cement. High values of AMD are not related to high values of microleakage probably because adhesive resin cements are resistant to dissolution and the hybrid layer allows a better seal and ensures adhesion and resistance to stresses [25], avoiding expression of microleakage; those findings agree with Rossetti et al. [13], who showed no strong correlation between margin fit parameters and microleakage. In our study, zirconia was not considered as adhesive substrate, showing cement as responsible for the sealing of the marginal gap without interaction between the restoration material and the dental surface. Castillo-Oyagüe et al. found a weak association between microleakage and AMD, showing similar results with respect to RelyX Unicem, with highest values for AMD and lowest for microleakage [26]. All the adhesive cements tested in Geerts et al.'s study showed small leakage scores demonstrating efficient results of these materials [23]. Wilson and Stankiewicz [21] showed no correlation among microleakage, cementation procedures, and different crown systems and speculate that the marginal fit is considered less important than the cement bonding combination used. In the present study the combination of zirconia coping with Rx Group presented the lowest value of microleakage; this finding agrees with Yüksel and Zaimoğlu [12], who obtained the lowest values with the same combination and related microleakage with the type of cement used. Though no correlation between AMD and microleakage was found, a logarithmic regression statistical model allowed us to find an association between these two variables (microleakage = AMD^0,896^).

## 5. Conclusions

Within the limitations of this study, the following conclusions can be drawn. (1) No significative microleakage and absolute marginal discrepancy differences were found between the 4 types of cements. (2) No linear correlations between microleakage and absolute marginal discrepancy were found; however, a logarithmic association between both variables could be deduced.

## Figures and Tables

**Table 1:** Microleakage and AMD values according to type of cement.

Type of cement groups	Microleakage area (*μ*m^2^)	AMD (*μ*m)
RelyX		
Mean	146,7392	264,4608
*N*	10	10
Dev	96,56190	29,75283

Maxcem		
Mean	166,0606	249,7805
*N*	10	10
Dev	97,86054	41,65023

Multilink		
Mean	180,8174	232,8893
*N*	10	10
Dev	111,17058	54,38182

PANAVIA		
Mean	252,7584	257,6856
*N*	10	10
Dev	170,36845	34,11377

Total		
Mean	186,5939	251,2040
*N*	40	40
Dev	124,73335	41,20680

*N*: sample; Dev: standard deviation; AMD: absolute marginal discrepancy.
